# The SmMYB36-SmERF6/SmERF115 module regulates the biosynthesis of tanshinones and phenolic acids in *salvia miltiorrhiza* hairy roots

**DOI:** 10.1093/hr/uhac238

**Published:** 2022-10-26

**Authors:** Qi Li, Xin Fang, Ying Zhao, Ruizhi Cao, Juane Dong, Pengda Ma

**Affiliations:** College of Life Sciences, Northwest A&F University, Yangling 71210, China; College of Life Sciences, Northwest A&F University, Yangling 71210, China; College of Life Sciences, Northwest A&F University, Yangling 71210, China; College of Life Sciences, Northwest A&F University, Yangling 71210, China; College of Life Sciences, Northwest A&F University, Yangling 71210, China; College of Life Sciences, Northwest A&F University, Yangling 71210, China

## Abstract

Tanshinone and phenolic acids are the most important active substances of *Salvia miltiorrhiza*, and the insight into their transcriptional regulatory mechanisms is an essential process to increase their content *in vivo*. SmMYB36 has been found to have important regulatory functions in the synthesis of tanshinone and phenolic acid; paradoxically, its mechanism of action in *S. miltiorrhiza* is not clear. Here, we demonstrated that SmMYB36 functions as a promoter of tanshinones accumulation and a suppressor of phenolic acids through the generation of *SmMYB36* overexpressed and chimeric *SmMYB36*-SRDX (EAR repressive domain) repressor hairy roots in combination with transcriptomic-metabolomic analysis. SmMYB36 directly down-regulate the key enzyme gene of primary metabolism, *SmGAPC*, up-regulate the tanshinones biosynthesis branch genes *SmDXS2*, *SmGGPPS1*, *SmCPS1* and down-regulate the phenolic acids biosynthesis branch enzyme gene, *SmRAS*. Meanwhile, SmERF6, a positive regulator of tanshinone synthesis activating *SmCPS1*, was up-regulated and SmERF115, a positive regulator of phenolic acid biosynthesis activating *SmRAS*, was down-regulated. Furthermore, the seven acidic amino acids at the C-terminus of SmMYB36 are required for both self-activating domain and activation of target gene expression. As a consequence, this study contributes to reveal the potential relevance of transcription factors synergistically regulating the biosynthesis of tanshinone and phenolic acid.

## Introduction

The different phenylpropanoid-derived compounds have a multitude of biological functions, including pigments, cell wall components, UV-absorbing compounds, phytoalexins, and modulators of developmental signaling. The MYB family of proteins is one of the largest transcription factor families and is regulator of phenylpropanoid synthesis in plants. Most subgroup 5 R2R3-MYB proteins are responsible for proanthocyanidin synthsis, most subgroup 6 R2R3-MYB proteins take part in anthocyanin synthesis, while the majority of R2R3-MYB subgroup 7 proteins contribute to flavonol synthesis. Most 1R-MYB and subgroup 4 R2R3-MYB proteins tend to be negative regulators of phenylpropanoid synthesis. MYB-bHLH-WD40 (MBW) complexes containing R2R3-MYB (subgroup 5 and 6), bHLH and WD40- repeat proteins regulate anthocyanin and proanthocyanidin synthsis [1, 2].

Terpenoids—including monoterpenes, sesquiterpenes, diterpenes, triterpenes, and tetraterpenes—play important roles as secondary metabolites in plant growth, development and protection in abiotic and biotic environments [3]. Overexpression of *Vitis vinifera VvMYB5b* increased β-carotene in tomato fruits and decreased its content in leaves [4]. *Citrus reticulate CrMYB68* repressed the transformation of α- and β-branch carotenoids [5]. The ectopic expression of *Arabidopsis AtPAP1* in rose flowers led to increased levels of terpenoid scent compounds, including monoterpenes, sesquiterpenes, and norisoprenes [6]. Constitutive overexpression of *Pinus taeda* subgroup 4 R2R3-MYB stimulatedsesquiterpene accumulation in conifers [7].

As a perennial medicinal plant, *Salvia miltiorrhiza* Bunge (also named Danshen) is a well-known member of the Labiatae family.Generally, phenolic acids and tanshinones are the primary bioactive components of *S. miltiorrhiza*, with phenolic acids produced by both the phenylpropanoid pathway and tyrosine-derivedpathways [8, 9]. As diterpenoids, tanshinones are synthesized bythe cytosolic mevalonate (MVA) and the plastidic 2-C-methyl-D-erythritol 4-phosphate (MEP) pathways. In recent years, genes thatare involved in tanshinones production, such as *SmDXR* (1-deoxy-Dxylulose −5-phosphate reductoisomerase) [10], *SmHMGR1* (3-hydroxy-3-methylglutaryl-CoA reductase) [11], *SmHMGR2* 30 [12], *SmIPI1* (isopentenyl diphosphate isomerase) [13], *SmGGPPS1* (geranylgeranyl diphosphate synthase) [11], *SmCPS1* (copalyl diphosphate synthase), *SmKSL1* (kaurene synthase-like) [14], *SmHMGR1*,*SmCYP76AH1* (cytochrome P450-dependent monooxygenase) [15], *SmCYP76AK1*, *SmCYP76AH3* [16], *SmCYP76AK2*, *SmCYP76AK3* [17], *Sm2OGD25* (Fe(II)/2-oxoglutarate-dependent dioxygenase) [18] and phenolic acids biosynthesis, such as *SmPAL1* (phenylalanine ammonia-lyase) [19], *SmTAT1* (tyrosine aminotransferase),*SmC4H1* (cinnamic acid 4-hydroxylase), *SmRAS* (rosmarinicacid synthase), *SmHPPR1* (4-hydroxyphenylpyruvate reductase), *SmCYP98A14* [20] have been characterized from *S. miltiorrhiza* [8–9]. Recently, there are some studies about tanshinone and phenolic acid regulation by transcription factors. SmERF115 is a negative regulator of tanshinone and promotes the accumulation of phenolic acid content by activating the expression of *SmRAS* [21]. SmERF6 directly co-regulates tanshinone biosynthesis by binding to the ethylene response element (GCC-box) of *SmKSL1* and *SmCPS1* promoters and activating their transcription to promote tanshinone biosynthesis [22]. SmMYB98 upregulated both tanshinone and phenolic acid biosynthesis [23], while SmMYB9b and SmMYB98b [24, 25] only up-regulated tanshinone biosynthesis. SmMYB1, SmMYB2, SmMYB97, SmMYB111 and SmPAP1 were positive regulators of salvianolic acid synthesis [26–28] and SmMYB39 is negative regulators [31]. Ternary transcription complex SmTTG1- SmMYB111-SmbHLH51was involved in the production of phenolic acid [25]. The mechanisms of regulation of the content of these MYB transcription factors for tanshinones and phenolic acids were mainly focused on the regulation of their secondary metabolic synthesis critical enzyme genes. SmMYB36 was the first R2R3-MYB transcription factor reported to regulate two active components of *S. miltiorrhiza*, enhancing the biosynthesis of tanshinones and reducing the accumulation of phenolic acids [32]. In short, SmMYB36 may well be used as a model to study the regulation of phenolpropane and terpene synthesis by the R2R3-MYB transcription factor.

In this study, we elucidated the regulatory functions of SmMYB36 on tanshinone and phenolic acid biosynthesis more deeply by generating *SmMYB36* overexpression and chimeric *SmMYB36*-SRDX repressor hairy roots and combining them with transcriptome-metabolome analysis. SmMYB36 was found to promote tanshinone synthesis and inhibit phenolic acid synthesis in three main regulatory ways: balance of primary and secondary metabolism; direct and indirect combination; and simultaneous positive and negative regulation. Additionally, the acidic amino acids of SmMYB36^154–160^ are required for the structural domains of transcriptional activation, also for the activation of target genes. Taken together, these results show the specific diversity, complexity, and regulatory gene expression network of SmMYB36 regulation of tanshinones and phenolic acids.

## Results

### SmMYB36 promoted the accumulation of tanshinone and inhibited the accumulation of phenolic acid in the hairy roots of *S. miltiorrhiza*

**Figure 1:**
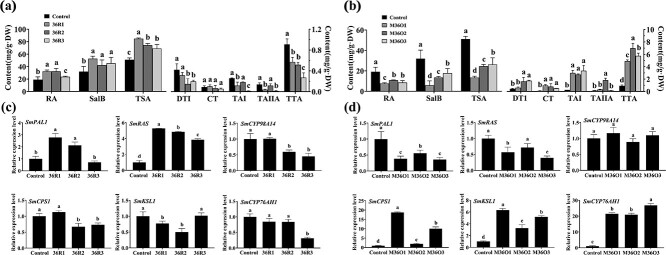
SmMYB36 promotes the accumulation of tanshinone and inhibits the biosynthesis of salvianoli in transgenic hairy roots. **a**, **b** The contents of DTI, CT, TAI, TAIIA, and TTA (**a**), the major components of tanshinone, and RA, SalB, and TPA (**b**), the salvianoli principal concentrations, in *SmMYB36* and *SmMYB36-SRDX* transgenic hairy roots. **c**, **d** Expression levels of *SmCPS1*, *SmKSL1*, and *SmCYP76AH1*, the key enzyme genes of tanshinone synthesis branch, and *SmPAL1*, *SmRAS*, and *SmCYP98A14*, the key enzyme genes of salvianoli synthesis branch. Empty ATCC15834 of transgenic hairy roots was used as control and error bars indicate standard deviation (SD) of three biological replicates. As determined by analysis of one-way and Duncan’s multiple range test (*P* < 0.05), different letters indicate significant differences.

To investigate the role of SmMYB36 in the synthesis of tanshinone and phenolic acid, pGWB18-Myc-*SmMYB36* (M36O) and pK7WG2R-*SmMYB36*-SRDX (36R) were transformed into hairy roots (Fig. S1a and b, see online supplementary material). The extracts of the M36O positive lines have a darker color than the control, while the 36R positive lines are essentially the same as the control (Fig. S1c, see online supplementary material). For the functional analysis of 36R hairy roots，the qRT-PCR results showed that *SmMYB36* expression in the three positive lines (36R1, 36R2, and 36R3) was 1.96-fold, 3.11-fold, and 3.77-fold higher than the control, respectively (Fig. S2, see online supplementary material). Compared with the control, the 36R positive lines showed significantly higher RA, Sal B, and TSA contents (*P* < 0.05), with an average increase of at least 1.6-fold, 1.5-fold, and 1.3-fold in RA, Sal B, and TSA contents (Fig. 1a). In contrast, the tanshinone components DTI, TA1, TAIIA, and TTA were significantly decreased in the 36R lines compared to the control (*P* < 0.05), while the CT content did not change significantly. Furthermore, as shown in Fig. 1c, phenolic acid biosynthesis pathway genes *SmPAL1* and *SmRAS* were up-regulated 2.12–5.27-fold, *SmCYP98A14* was down-regulated about 0.45–0.59-fold, and tanshinone biosynthesis pathway genes *SmCPS1*, *SmKSL1*, and *SmCYP76AH1* were down-regulated 0.31–0.77-fold, respectively, compared with the control.

For the functional analysis of M36O hairy root, qRT-PCR analysis revealed that *SmMYB36* expression was 2.5-, 3.5-, and 7.3-fold higher in the overexpression lines M36O1, M36O2, and M36O3 than the control (Fig. S2, see online supplementary material). Compared with the control lines, the content of RA, SalB, and TSA in the M36O lines was significantly lower (*P* < 0.05), and all could be decreased by up to more than 50% (Fig. 1b). The expression of *SmPAL1* and *SmRAS*, key genes of the phenolic acid biosynthesis pathway, decreased approximately 36%–57%, whereas *SmCYP98A14* showed no significant difference compared to the control. In addition, the tanshinone biosynthesis pathway genes *SmCPS1*, *SmKSL1*, and *SmCYP76AH1* were increased by around 1.90–18.77-fold, 3.26–6.35-fold, and 20.92–26.77-fold, respectively (Fig. 1d). Consequently, these results suggest that SmMYB36 functions as a positive regulator of tanshinone biosynthesis a negative regulator of phenolic acid biosynthesis.

### Overexpressing *SmMYB36* altered different primary and secondary metabolites by widely targeted metabolic profiling

To investigate the metabolic changes in the *SmMYB36* overexpression line compared with the control line, widely targeted metabolome was applied. A total of 327 metabolites were detected, including carbohydrates, amino acid and derivatives, mucleotide and derivates, vitamins and derivatives, organic acids and derivative, lipids, alcohols, terpenes, phenolamides, quinones, alkaloids, indole derivatives, sterides, phenolamides, phenylpropanoids, anthocyanin, flavone, flavonoid, flavonol, isoflavone, polyphenol, *et al.* (Table S3, see online supplementary material).

Then, comparing the *SmMYB36* overexpression line with control line, there were 142 differential metabolites, of which 109 were down-regulated and 33 were up-regulated (Table S4, see online supplementary material). In this study, 26 differential amino acid and derivatives (23 down and 3 up) were identified, specially including nine down-regulated standard amino acds (L-histidine, L-glutamine, L-proline, L-asparagine, L-phenylalanine, L-alanine, L-tyrosine, L-leucine, L-isoleucine). Succinic acid in TCA cycle was down-regulated. Twenty-four differential phenylpropanoid metabolites (21 down and 3 up) were identified, including 2 anthocyanins (2 down), 2 flavones (1 down and 1 up), 3 flavonols (3 down), 2 isoflavones (1 down and 1 up), 1 polyphenol (1 down), and 14 unclassified phenylpropanoids (13 down and 1 up). In view of the fact that the content of RA and SalB was significantly reduced in *SmMYB36* transgenic lines, overexpression of *SmMYB36* decreased phenylpropanoid production in *S. miltiorrhiza*. Four differential terpenoid metabolites were identified, including one monoterpenoid (geniposidic acid), two sesquiterpenoids (roseoside, β-caryophyllene), one diterpenoid (phytol), of which all were down-regulated. Considering that the content of DTI, CT, TA1, and TAIIA was also significantly increased in 36O lines, overexpression of *SmMYB36* increased diterpenoid yield in *S. miltiorrhiza*. As a result, these results also suggest that SmMYB36 up-regulates tanshinone content and down-regulates phenolic acid content by influencing the synthesis of various primary and secondary metabolites.

### Overexpressing *SmMYB36* altered different primary and secondary metabolite parthway biosynthesis enzyme gene by transcriptome profiling

To better analyse the potential roles of SmMYB36 in primary andsecondary metabolic regulation, we conducted transcriptomeprofiling for the *SmMYB36* overexpression line compared with the control line. Compared to control lines, a total of 6726 DEGs were detected in the *SmMYB36* overexpression line,which was comprised of 3583 genes up-regulated and 3143 genes downregulated. All the DEGs were annotated according to GO, KEGG, KOG, Nr, Swiss-Prot, and TrEMBL (Table S5, see online supplementary material). These DEGs were assigned to three classes of GO: molecular functions, cellular compartment, and biological process. The GO terms ‘cellular process’, ‘metabolic process’ and ‘response to stimulus’ are the most highly enriched among biological process (Fig. S3a, see online supplementary material). KEGG analysis showed that ‘metabolic parthways’, ‘biosynthesis of secondary metabolites’ and ‘phenylpropanoid’ were significantly enriched (Fig. S3b, see online supplementary material). KOG analysis indicated that ‘signal transduction mechanisms’, ‘secondary metabolites biosynthesis, transport, and catabolism’, ‘post-translationalmodification, protein turnover, chaperones’ and were significantly enriched (Fig. S3c, see online supplementary material).

**Figure 2:**
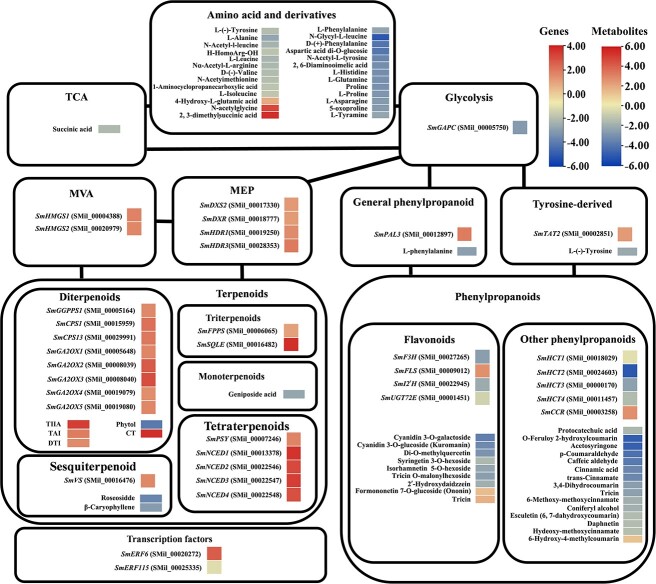
Transcriptome combined with metabolome analysis in *SmMYB36* overexpressed hairy roots. The wide heat map scale represents the relative level of metabolites and the narrow heat map scale represents the relative level of gene expression.

As shown in Fig. 2, among primary metabolite parthway biosynthesis enzyme genes, the expression of transcripts coding for glycolysis enzymes such as glyceraldehyde 3-phosphate dehydrogenase (*SmGAPC*) (SMil_00005750) was reduced.

Among phenylpropanoid biosynthesis enzyme genes, thegenes coding for *SmPAL3* (SMil_00012897) in general phenylpropanoid parthway and tyrosine aminotransferase 2 (*SmTAT2*) (SMil_00024925) in tyrosine-derived parthway was up-regulated.Among flavonoid biosynthesis enzyme genes, the transcripts coding for flavonoid-3-hydroxylase (*SmF3H*) (SMil_00027265), anthocyanidin isoflavone 2′-hydroxylase (*SmI2’H*) (SMil_00022945) andconiferyl-alcohol glucosyltransferase (*SmUGT72E*) (SMil_00001451) were down-regulated, while the transcript coding for flavonol synthase (*SmFLS*) (SMil_00009012) was upregulated. Among other phenylpropanoid biosynthesis enzyme genes, the transcripts coding for shikimate hydroxycinnamoyl transferase (*SmHCT1/2/3/4*) (SMil_00018029, SMil_00024603, SMil_00000170, SMil_00011457) were down-regulated, while the transcripts coding for cinnamoyl-CoA reductase (*SmCCR*) (SMil_00003258) were up-regulated.

Among terpenoid biosynthesis enzyme genes, a gene coding for 1-deoxy-D-xylulose-5-phosphate synthase 2 (*SmDXS2*)(SMil_00017330), 1-deoxy-D-xylulose 5-phosphate reductoisomerase (*SmDXR*) (SMil_00018777), hydroxy-2-methyl-2-(E)-butenyl-4-diphosphate reductase1, 3(*SmHDR1*, 3) (SMil_00019250, SMil_00028353) in the MEP pathway was up-regulated. Among MVA pathway genes, the transcripts coding for hydroxymethylglutaryl-CoA synthase1/2 (*SmHMGS1/2*) (SMil_00004388, SMil_00020797) were upregulated. The genes for vetispiradiene synthase (*SmVS*) (SMil_00016476) in sesquiterpenoid; *SmGGPPS1* (SMil_00005164), *SmCPS1*, 13(SMil_00015959, SMil_00029991), gibberellin 2-beta-dioxygenase1/2/3/4/5 (*SmGA2OX1/2/3/4/5*) (SMil_00005648, SMil_00008039, SMil_00008040, SMil_00019079,SMil_00019080) in diterpenoid; farnesyl diphosphate synthase(*SmFPPS*) (SMil_00006065) and squalene monooxygenase (*SmSQLE*) (SMil_00016482) in triterpenoid; phytoene synthase (*SmPSY*) (SMil_00007246), 9-cis-epoxycarotenoid dioxygenase1/2/3/4 (*SmNCED 1/2/3/4*) (SMil_00013378) (SMil_00022546)(SMil_00022547) (SMil_00022548) in tetraterpenoid biosynthesis enzyme genes were increased.

In addition, two transcription factor genes involving in tanshinone and salvianolic acids regulation were detected by transcriptome profiling. *SmERF6* (ethylene responsive factor) (SMil_00020272) was up-regulated and *SmERF115* (SMil_00025335) was down-regulated.

To validate the transcriptome profiling results, 10 DEGs were chosen for qPCR analysis, including two transcription factor genes, 2 phenylpropanoid biosynthetic pathway genes, and 6 flavonoid biosynthetic pathway genes (Fig. S4). Consistently, the qRT-PCR results follow the same trend as the transcriptome sequencing data.

### SmMYB36 acts directly on *SmGAPC*, *SmGGPPS1*, *SmCPS1*, *SmRAS*, *SmDXS2*, *SmERF6* and *SmERF115* to synergistically regulate the accumulation of tanshinones and phenolic acids

**Figure 3:**
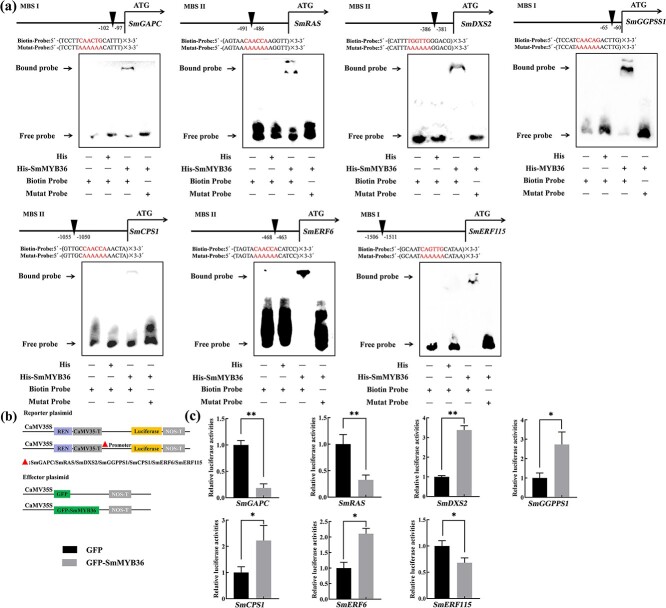
SmMYB36 binds directly to specific motifs in the promoters of *SmGAPC*, *SmGGPPS1*, *SmCPS1*, *SmRAS*, *SmDXS2*, *SmERF6*, and *SmERF115* and promotes the expression of *SmGGPPS1*, *SmCPS1*, *SmDXS2*, *SmERF6* and represses the expression of *SmRAS*, *SmERF115*, and *SmGAPC* expression. **a** Results of EMSA experiments. **b**–**c** Schematic diagram of dual-LUC vector construction (**b**) and experimental results (**c**). The LUC/REN activities of the experimental plasmid combinations (*SmGAPCpro*::LUC + GFP-SmMYB36, *SmGGPPS1pro*::LUC + GFP-SmMYB36, *SmCPS1pro*::LUC + SmMYB36-GFP, *SmRASpro*::LUC+ GFP-SmMYB36, *SmDXS2pro*::LUC + SmMYB36-GFP, *SmERF6pro*::LUC+ GFP-SmMYB36, and *SmERF115pro*:: LUC + SmMYB36-GFP) and the corresponding control plasmid combinations (*SmGAPCpro*::LUC + GFP, *SmGGPPS1pro*::LUC + GFP *SmCPS1pro*::LUC + GFP, *SmRASpro*::LUC + GFP, *SmDXS2pro*::LUC + GFP, *SmERF6pro*::LUC + GFP, and *SmERF115pro*::LUC + GFP) were measured and analysed, respectively. Error bars represent the SD (Student’s *t*-test, ^*^P < 0.05, ^**^P < 0.01).

To verify whether SmMYB36 acts directly on genes or regulators of tanshinone and phenolic acid biosynthesis, in vitro experiments EMSA were performed. As shown in Fig. 3a, SmMYB36 binds to the 3 × MBSI/II core motif probes in *SmGAPC*, *SmRAS*, *SmDXS2*, *SmGGPPS1*, *SmCPS1*, *SmERF6* and *SmERF115*, but not to the mutation probes.

**Figure 4:**
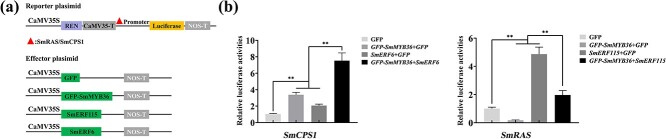
SmMYB36 and SmERF6 cooperatively and positively regulate *SmCPS1* expression, and SmMYB36 and SmERF115 antagonistically regulate *SmRAS* expression. **a** Schematic diagram of reporter vector containing *SmRAS* and *SmCPS1* promoter and effector vector containing GFP-SmMYB36, SmERF115, and SmERF6 (**b**). LUC/REN activity was measured and comparatively analysed for co-transformation of different combinations of effector and reporter plasmids *SmCPS1pro*::LUC + GFP, *SmCPS1pro*::LUC + GFP-SmMYB36 + GFP, *SmCPS1pro*::LUC+ SmERF6 + GFP, *SmCPS1pro*::LUC + GFP-SmMYB36 + SmERF6, *SmRASpro*::LUC + GFP, *SmRASpro*::LUC + GFP-SmMYB36 + GFP, *SmRASpro*::LUC + SmERF115 + GFP, and *SmRASpro*::LUC + GFP-SmMYB36 + SmERF115. Error bars represent the SD (Student’s *t*-test, ^*^P < 0.05, ^**^P < 0.01).

To validate that SmMYB36 is acting as a regulator of target genes, *in vivo* experiments were performed with dual-LUC. *SmGAPC*, *SmRAS*, *SmDXS2*, *SmGGPPS1*, *SmCPS1*, *SmERF6*, and *SmERF115* promoters drive the *LUC* gene to form a reporter, and *SmMYB36* was overexpressed as an effector in the presence of the 35S promoter. LUC luminescence assays showed that co-expression with *SmMYB36* increased the expression of *SmDXS2pro*::LUC, *SmGGPPS1pro*::LUC, *SmCPS1pro*::LUC, and *SmERF6pro*::LUC reporter genes, and declined the expression of *SmGAPCpro*::LUC, *SmRASpro*::LUC, and *SmERF115pro*::LUC expression, compared to controls lacking *35Spro*::SmMYB36 (Fig. 3b). Further analysis of the mechanism of SmMYB36-SmERF6/SmERF115 co-regulation of *SmCPS1* and *SmRAS* using dual-LUC assay also supported the finding that SmMYB36 and SmERF6 together enhanced the expression of *SmCPS1*, and SmMYB36 and SmERF115 acted antagonistically on the regulation of *SmRAS* (Fig. 4a and b). Accordingly, these results suggest that SmMYB36 is a positive regulator of tanshinones biosynthesis and a negative regulator of phenolic acids synthesis.

### The C-terminus of SmMYB36 has an acidic region that is required for transcriptional activation and activation of target genes

**Figure 5:**
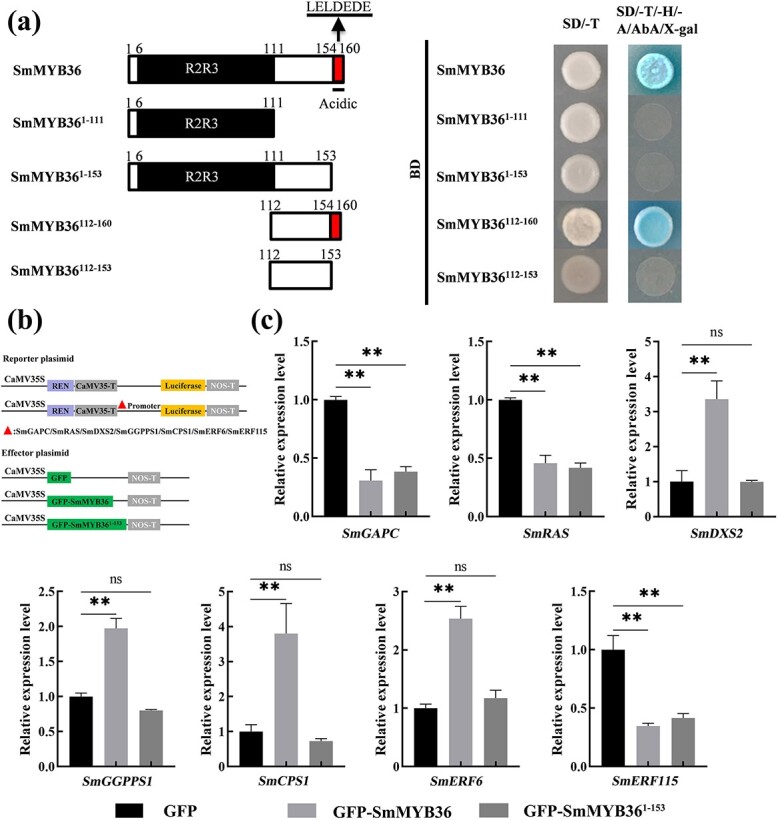
SmMYB36^154–160^ is both a self-activating structural domain and a region that activates the expression of *SmGGPPS1*, *SmCPS1*, *SmDXS2*, and *SmERF6*. **a** Analysis of the SmMYB36 self-activating structural domain in yeast. **b** Schematic diagram of the construction of the reporter plasmid and the effector plasmid. **c** Dual-LUC analysis of the effect of SmMYB36^1–153^ on the expression of *SmGAPC*, *SmRAS*, *SmDXS2*, *SmGGPPS1*, *SmCPS1*, *SmERF6*, and *SmERF115* genes. Error bars indicate the standard error of the mean of three experimental replicates (*P* < 0.05, *P* < 0.01).

To identify the specific location of the transcriptional activation domain of SmMYB36, we constructed its truncated pGBKT7 bait vector. The truncated SmMYB36 containing the C-terminal acidic region SmMYB36^153–160^ was transcriptionally active, and the rest (SmMYB^1–111^, SmMYB36^1–153^, SmMYB36^112–153^) lost transcriptional activity (Fig. 5a).

To confirm whether the transcriptional activation domain of SmMYB36 is associated with the activation of target genes, *SmMYB36^1–153^* was constructed as the effector vector for dual-LUC experiments (Fig. 5b). It was found that compared with SmMYB36, SmMYB36^1–153^ lost its ability to activate positively regulated target genes, but its effect on repressing target genes was still retained.

**Figure 6:**
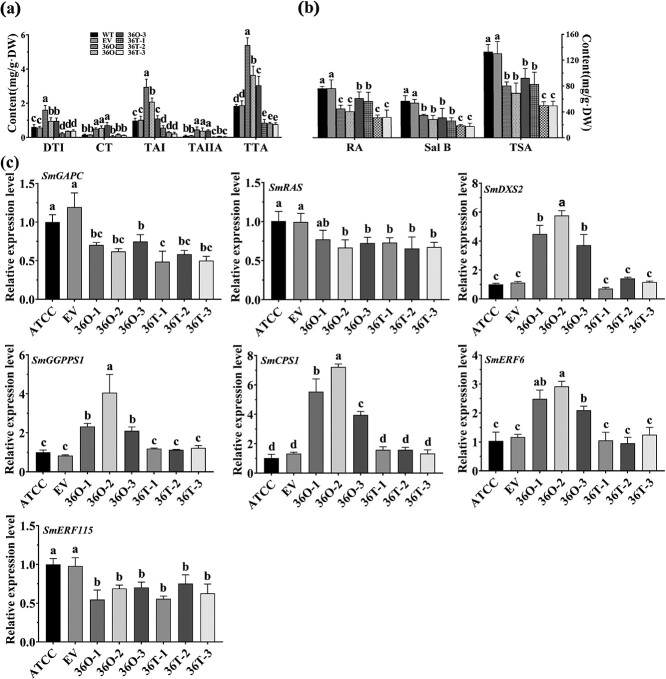
SmMYB36^1–153^ decreased the promoting effect of SmMYB36 on the accumulation of total tanshinone in *S. miltiorrhiza* hairy root. **a**, **b** The contents of DTI, CT, TAI, TAIIA and TTA, the major components of tanshinone, and RA, SaIB and TPA, the phenolic acids principal concentrations, in *SmMYB36 and SmMYB36^1–153^* transgenic hairy roots. **c** Expression levels of SmMYB36 target, genes *SmGAPC*, *SmRAS*, *SmGGPPS1*, *SmCPS1*, *SmRAS*, *SmDXS2*, *SmERF6*, and *SmERF115.* Empty ATCC15834 (ATCC) and empty vector (EV) of transgenic hairy roots was used as control and error bars indicate standard deviation (SD) of three biological replicates. A different letter indicates a significant difference between the groups, according to the analysis of one-way ANOVA and Duncan’s multiple range test (*P* < 0.05).

To further determine the function of amino acids at the C-terminus of SmMYB36 in *S. miltiorrhiza* endogenous, transgenic hairy roots of *SmMYB36* (36O) and *SmMYB36^1–153^*(36 T) were obtained. As shown in Fig. S5b and c (see online supplementary material), the color of the control and 36 T transgenic hairy roots and extracts was lighter compared to that of 36O. Three lines with 3–7-fold higher *SmMYB36* expression levels than the controls (ATCC and EV) were selected separately for follow-up experiments (Fig. S6, see online supplementary material). The results of the HPLC assay showed that compared with the overexpression lines of *SmMYB36* (36O-1, 36O-2, and 36O-3), the overexpression lines of *SmMYB36^1–153^* (36 T-1, 36 T −2, and 36 T-3) showed lower levels of tanshinones (DTI, CT, TA1, TAIIA, and TTA) and their biosynthesis-related target genes (*SmDXS2*, *SmGGPPS1*, *SmCPS1*, and *SmERF6*), while the levels of phenolic acids (RA, SalB, and TSA) and biosynthesis-related target genes (*SmGAPC*, *SmRAS*, and *SmERF115*) were largely unchanged (Fig. 6a–c). These results suggest that the acidic region at the C-terminus of SmMYB36 is responsible for the activating effect of the target gene, but is not related to its repressive function.

## Discusssion

### SmMYB36 acts as both activator and repressor

In this study, EMSA and Dual-luc assays revealed that SmMYB36 specifically binds to *cis*-acting elements of *SmDXS2*, *SmGGPPS1*, *SmCPS1*, and *SmERF6* promoter regions and increases their expression levels, and also binds to specific DNA motifs of *SmGAPC*, *SmRAS*, and *SmEFR115* but decreases their expression levels. These results suggest that SmMYB36 acts as both a transcriptional activator and a repressor. In truth, there are many studies that found that R2R3-MYB transcription factors mainly regulate the transcriptional levels of target genes as activators, with the majority of repressors belonging to subgroup 4 (SG4) [1, 2]. It is highly conserved that the N-terminal end of R2R3-MYB TFs contains a functional DNA binding domain (MYB domain), while the C-terminal end possesses a variable activation or repression domain. Although we have identified that SmMYB36 has transactivation activity [32], the exact location and specific sequence of its activation domain are still unclear. Previously, it has been identified that functional MYB transcription factors such as GL1 (GLABRA1), WEREWOLF (WER), AtMYB23 and AtMYB2 contain a segment of acidic amino acid residues at their C-terminus that are required for transcriptional activation [36, 37]. Similarly, an acidic amino acid residue (LELDEDE) was identified at the C-terminal 154 to 160 amino acids of SmMYB36, and we also demonstrated that it plays a decisive role in the transcriptional regulation of downstream target genes. Additionally, it is believed that the bHLH can interact with R2R3-MYB to enhance the transcriptional activation of R2R3-MYB [1]. SmMYB36 also contains a bHLH-interacting motif in the R3 structural domain ([D/E] Lx2[R/K]x3Lx6Lx3R), but the bHLH that interacts with it and the effect on its transcriptional activity has not been reported. The SG4 R2R3-MYB repressors is known to have a ‘C2’ repressor motif at the C-terminus containing an EAR motif core sequence (LXLXL), for which the mechanism of EAR action is not clear. In other repressor proteins, the EAR pattern functions by recruiting co-repressors [38]. It is puzzling that SmMYB36 does not belong to SG4 of the R2R3-MYB transcription factors and does not contain an EAR motif at the C-terminus, so it will be interesting to explore the mechanism of how it functions as a repressor. We speculate that SmMYB36 may have an unknown transcriptional repressor domain, and another possibility is that it can recruit other repressors to inhibit the transcription of target genes. For instance, two R2R3-MYB, GL1 (GLABRA1) and MYB75, belonging to the MBW complex, are able to interact with the recruitment of repressor JAZ proteins thereby inhibiting JA-mediated trichome initiation and anthocyanin accumulation [39]. The JAZ repressor also interacts with MYB21 and MYB24, which are required for stamen development, and represses their transcriptional activity [20]. The repression activity of AtMYB3 to phenylpropanoid biosynthesis enzyme cinnamate 4-hydroxylase (C4H) gene expression was enhanced via interaction with co-repressors AtLNK1 (NIGHT LIGHT-INDUCIBLE AND CLOCK-REGULATED1) and AtLNK2 [40]. Therefore, the further studies could focus on identifying the transcriptional repression structural domains of SmMYB36 C-terminus, or focus on screening SmMYB36 interaction co-activators and co-repressors to deepen the mechanism of SAMMYB36 activators and repressors.

### SmMYB36 coordinately regulates the primary and secondary metabolic pathway

Recently, large-scale yeast monohybrid experiments have demonstrated that most transcription factors in plants bind to the promoters of multiple metabolic pathway genes, instead of binding specifically to the promoter of a particular pathway gene. In general, different transcription factors can co-regulate different metabolic pathways [41]. It is well known that the products of plant primary metabolism provide energy and raw materials for secondary metabolism through glycolysis and tricarboxylic acid cycle (TAC). Previous studies have indicated that the metabolic flux of phenylpropanoid biosynthesis is increased by overexpression of the 3-deoxy-D-arabinoheptulose 7-phosphate synthase (*DAHPS*) gene, which enhances the activity of the shikimate pathway [42, 43]. Homoplastically, AtMYB12 not only activated the expression of enolase (*ENO*) in primary metabolic enzyme genes in glycolysis and *DAHPS* gene in the shikimate pathway, but also activated flavonoid enzyme genes to drive carbon flux toward phenylpropanoid biosynthesis [44]. Overexpression of *SlMIXTA*-like can increase phenylpropanoid content by binding to the promoter region of the gene encoding *DAHPS*, thereby increasing the flux of downstream secondary metabolites [45]. Silencing of mitochondrial citrate synthase gene *PhmCS* or ATP-citrate lyase genes *PaACLA1-A2* and *PaACLB1-B2* reduced total anthocyanin content in petunia [46, 47]. As a consequence, regulating the expression of primary metabolic genes is an effective way to alter the accumulation of secondary metabolites. In the present study, SmMYB36 regulated both *SmGAPC* in the primary metabolic glycolytic pathway and *SmRAS* gene in the secondary metabolic phenolic acid synthesis and *SmDXS2*, *SmGGPPS1*, and *SmCPS1* genes in the tanshinone synthesis. In the glycolytic pathway, GAPC catalyzes the conversion of 3-phosphoglycerate to 1,3-diphosphoglycerate. *GAPC* deficient mutant line shows a decrease in ATP levels and reduced levels of pyruvate and several TAC intermediates in Arabidopsis [48]. Of note, our results showed that down-regulation of *SmGAPC* by SmMYB36 results in less succinate in the Krebs cycle and reduced biosynthesis of phenylalanine and tyrosine, the precursors of salvianolic acids synthesis. This is detrimental to the availability of substances and energy for the synthesis of phenolic acids. In combination with the fact that SmMYB36 down-regulated the expression of *SmRAS*, a key enzyme for phenolic acid synthesis, which further exacerbated the decrease in the accumulation of phenolic acid synthesis. Taken together, the down-regulation of phenolic acid accumulation in *SmMYB36* overexpressing hairy roots is a result of the superimposed reduction in primary and secondary metabolism. Alternatively, for the increased accumulation of tanshinones in *SmMYB36* overexpressing hairy roots was mainly attributed to the positive regulation of secondary metabolism key enzymes *SmDXS2*, *SmGGPPS1*, and *SmCPS1* by SmMYB36, while the negative regulation of *SmGAPC* by SmMYB36 was clearly detrimental to its accumulation.

### SmMYB36 coordinately directly and indirectly regulate tanshinone and salvianolic acid biosynthesis enzyme genes

SmMYB36 was shown to bind and activate *SmERF6* and *SmCPS1* promoters by EMSA and dual-LUC. Previous studies have demonstrated that SmERF6 binds to the GCC-box of the *SmCPS1* promoter by EMSA and yeast one-hybrid (Y1H) and that it acts as an activator of tanshinone analog synthesis [22]. These results suggest that SmMYB36 positively regulates the accumulation of tanshinones directly or indirectly through SmERF6. We demonstrated by EMSA and dual-LUC that SmMYB36 binds to *SmERF115* and *SmRAS* promoters to repress their expression. It has been previously demonstrated by yeast one-hybrid, EMSA and dual-LUC that SmERF115 binds and activates the GCC-box of the *SmRAS* promoter and that it acts as an activator of phenolic acid synthesis [21]. Thus, SmMYB36 negatively regulates the accumulation of phenolic acids directly or indirectly through SmERF115. Known as a feed-forward loop, this hierarchical regulatory network consists of one transcription factor regulating another transcription factor, which in turn co-regulates downstream targets [49]. Just as, in a multi-level feed-forward loop, MYB46/MYB83 coordinates with their regulators, secondary wall NACs (SWNs) proteins and their direct targets regulate a series of downstream target genes and secondary wall formation in *Arabidopsis* [50–52]. Particularly, this type of feed-forward loop regulatory mechanism has been reported in phenylpropanoid and terpenoid regulation. In the biosynthesis of artemisinin, *AaMYC2* and *AabZIP1* can be regulated by forming a bifurcated feedforward loop with TRICHOME-SPECIFIC WRKY 1 (AaGSW1), respectively [53]. The robustness of the signaling process can be improved by this coherent feed-forward loop of regulation [54]. In general, SmMYB36 further enhanced the positive and negative regulatory effects on tanshinones and phenolic acids through this feed-forward loop regulation.

### Mechanism of SmMYB36 positively and negatively regulating the synthesis of tanshinones and phenolic acids

**Figure 7:**
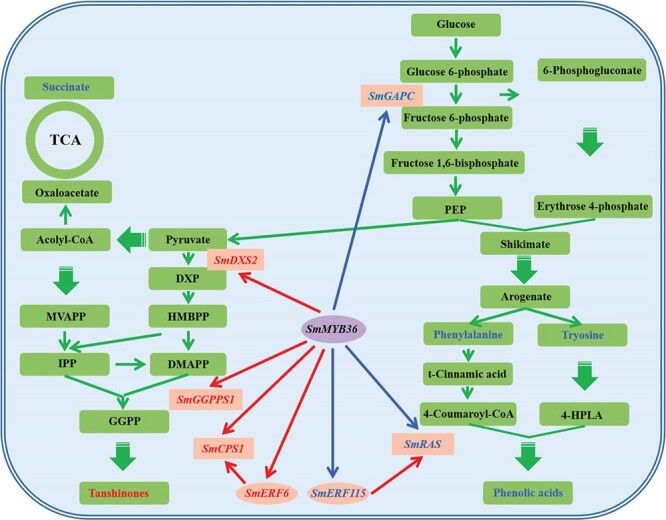
Schematic representation of SmMYB36 regulation of tanshinone and phenolic acid biosynthesis. The narrow solid arrows indicate single-step or proven reactions, while the wide dashed arrows represent multi-step or speculative reaction processes. Red font and arrows represent metabolites or genes that are positively regulated, blue arrows and font represent metabolites or genes that are negatively regulated.

Transcription factors regulating the synthesis of tanshinone and phenolic acid in *S. miltiorrhiza* have been reported extensively. SmMYC2a (SmMYC2), SmMYC2b and SmMYB98 as positive regulators and SmbHLH3 as negative regulators, while SmERF1L1, SmERF115, SmGRAS1, SmGRAS2 and SmbZIP1 are positive and negative bidirectional regulators [21, 35, 55–60]. Studies on the mechanisms of regulation of tanshinone and phenolic acid by these transcription factors have focused on the regulation of key enzyme genes of the downstream synthetic pathway. Nonetheless, few studies have explored the mechanisms of primary metabolism level regulation and feed-forward loop regulation. Yang *et al.* (2017) [57] demonstrated by transcriptome that overexpression of *SmMYC2* upregulated genes in the mangiferous acid synthesis pathway, but the necessary experimental evidence is lacking as to whether SmMYC2 directly initiates the expression of these genes and whether the upregulated expression of these genes enhances the accumulation of phenylalanine and tyrosine. Consequently, it cannot be ruled out that SmMYC2 has a direct up-regulatory effect on downstream genes of key enzymes for phenolic acid synthesis and forms a ‘pull’ on the upstream manganate pathway, rather than a simultaneous direct regulation of primary and secondary metabolic pathways by SmMYC2. Deng *et al.* (2020) [59] showed that SmbZIP1 negatively regulated *SmERF1L1*, and the two formed a feed-forward loop to positively regulate the accumulation of tanshinones and phenolic acids. It can be understood that SmERF1L1 acts as a positive regulator of tanshinones and a negative regulator of phenolics, and SmbZIP1 inhibits the expression of phenolics negative regulators and thus promotes phenolics accumulation. It is puzzling that SmbZIP1 inhibited the positive regulator of tanshinones and the result of SmbZIP1 as the positive regulator of tanshinones is contrary. This suggests that there may be some complex regulatory mechanisms of SmbZIP1 as a positive regulator of tanshinones that have not been revealed. Hence, we found that SmMYB36 coordinates the regulation of tanshinone and phenolic acid through three levels: it acts as both activator and repressor in the first level; coordinates the regulation of primary and secondary metabolic pathways in the second level; and coordinates the regulation of tanshinone and phenolic acid biosynthetic enzyme genes directly and indirectly in the third level. Unexpectedly, the regulatory mechanisms for SmMYB36 specifically for the accumulation of tanshinone and phenolic acid are still remarkably different. SmMYB36 enhances tanshinone biosynthesis in *S. miltiorrhiza* through two mechanisms: (i) SmMYB36 initiated the transcription of *SmDXS2*, *SmCPS1*, and *SmGGPPS1* to directly promote tanshinone biosynthesis; and (ii) SmMYB36 promotes the expression of *SmERF6*, the activator of tanshinone biosynthesis, and indirectly promotes tanshinone biosynthesis. Moreover, SmMYB36 decreased salvianolic acid biosynthesis in *S. miltiorrhiza* through three directions: (i) SmMYB36 directly down-regulated *SmGAPC* expression, which directly reduced accumulation of phenylalanine and tyrosine; (ii) SmMYB36 directly down-regulated *SmRAS* transcription abundance and thus directly inhibited phenolic acid synthesis; and (iii) SmMYB36 indirectly inhibited phenolic acid synthesis by suppressing *SmERF115* expression, an activator of phenolic acid synthesis (Fig. 7).

In conclusion, the synthesis of phenolic acids was comprehensively inhibited by SmMYB36 from primary to secondary, and from direct to indirect; whereas the synthesis of tanshinones was positively regulated by SmMYB36 only for secondary metabolic pathways, both directly and indirectly. Our results fully demonstrate that SmMYB36 is functionally pleiotropic in regulating the synthesis of tanshinone and phenolic acid substances, one transcription factor acts on multiple pathways to exert multiple effects, which lays the foundation for a comprehensive understanding of the regulatory mechanism of the active ingredients of *S. miltiorrhiza*, and provides a useful tool for the future use of genetic engineering to improve the quality of *S. miltiorrhiza* and precisely regulate the active ingredients.

## Materials and methods

### Plasmid construction

For constructing the hairy root genetic transformation vector, the complete open reading frame (ORF) sequence of *SmMYB36* and its C-terminus containing SRDX (LDLDLELRLGFA) nucleotide sequence were cloned separately by PCR amplification and constructed onto the entry vector pDNOR207 using BP clonase enzyme. After that, the target sequences on the entry vector were constructed on pGWB18 and pK7WG2R using LR clonase enzyme to obtain pK7WG2R-*SmMYB36*-SRDX and pGWB18-Myc-*SmMYB36*, respectively. For transcriptional activation experiments, the amplified fragments (*SmMYB3*6^1–160^, *SmMYB36*^1–111^, *SmMYB36*^1–153^, *SmMYB36*^112–160^, and *SmMYB36*^112–153^) were constructed into the pDONR207 entry vector and pDEST-GBKT7 destination vector, separately and sequentially, using Gateway technology. For the functional characterization of SmMYB36^1–153^ in transgenic hairy roots, the coding sequences (CDS) of SmMYB36 and SmMYB36^1–153^ (LELDEDE with seven amino acids removed) were constructed into the pK7WG2R vector using Gateway technology. The above vector construction method was performed using gateway technology and the procedure follows the instructions of the BP and LR Clonase Kits (Invitrogen, USA).

For dual-LUC experiments, promoters of *SmGAPC*, *SmRAS*, *SmDXS2*, *SmGGPPS1*, *SmCPS1*, *SmERF6*, and *SmERF115* were cloned and constructed into the pGreenII 0800-LUC vector as reporter vectors. The sequences of *SmMYB36* and *SmMYB36^1–153^* were cloned into pCsGFPBT, and the full length of the ORFs of *SmERF6* and *SmERF115* were cloned into pGreenII 62-SK as effector vectors. The vector was constructed by a double digestion method according to the instructions of Clon Express MultiS One Step Cloning Kit (Vazyme Biotech, China). For EMSA assays, the CDS of SmMYB36 was constructed into the pET32a vector to obtain the fusion expression vector pET32a-His-*SmMYB36*. The gene sequence information involved in this study was obtained from the National Genome Data Center (https://ngdc.cncb.ac.cn/search/) under the accession number GWHAOSJ00000000. All primers involved in the above vector construction process are shown in Table S1 (see online supplementary material).

### Transcriptional activation domain analysis

To identify the transcriptional activation domain of SmMYB36, pDEST-GBKT7-*SmMYB36^1–160^*, pDEST-GBKT7-*SmMYB36^1–111^*, pDEST-GBKT7-*SmMYB36^1–153^*, pDEST-GBKT7-*SmMYB36^112–160^*, and pDEST-GBKT7- *SmMYB36^153–160^* plasmids were grown on defective medium SD/−Trp after transformation into yeast strain Y2HGold. The pDEST-GBKT7 plasmid was used as control. Subsequently, the transformed yeast cells were inoculated on SD/−Trp/-His/−Ade/X-α-gal/AbA (200 ng/ml) medium using the pDEST-GBKT7 plasmid as a control to observe the results.

### Plant transformation

The plasmids pGWB18-Myc-*SmMYB36* (M36O), pK7WG2R-*SmMYB36*-SRDX (36R), pK7WG2R-*SmMYB36* (36O), and pK7WG2R-*SmMYB36^1–153^* (36 T) were transformed with *Agrobacterium rhizogenes* ATCC15834 to induce *S. miltiorrhiza* hairy roots, ATCC15834 (ATCC) and transformants containing empty vector (EV) induced hairy roots and served as controls. Positive lines of transgenic hairy roots were validated using the reported method [32].

### Quantitative real-time PCR (qRT-PCR)

With the SteadyPure plant RNA extraction kit (ACCURATE, China), total RNA was extracted from transgenic hairy roots (M36O, 36R, 36O, and 36 T) and converted to cDNA with EvoM-MLV reverse transcription kit (ACCURATE). The qRT-PCR was performed following the previous method [32]. Three biological repetitions were conducted. All primers used in the qRT-PCR are found in Table S2 (see online supplementary material).

### High-performance liquid chromatography (HPLC)

The dried hairy root samples of M36O, 36R, 36O, and 36 T transgenic and control lines were powdered with a mortar and pestle, weighed precisely 0.02 g in a 10 mL centrifuge tube, extracted with 4 mL of 70% methanol overnight and then treated with ultrasonic shaking for 45 min, centrifuged at 10000 rpm for 10 min at room temperature, the supernatant was removed and stored at 4°C on a 0.45 μm filter membrane. As described previously, HPLC analysis was performed on three biological replicates of every sample [31]. Total phenolic acid (TPA) was determined by the sum of SalB (salvianolic acid B) and RA (rosmarinic acid), and the sum of tanshinone IIA (TIIA), while cryptotanshinone (CT), dihydrotanshinone I (DTI) and tanshinone I (TAI) was considered as total tanshinone (TTA).

### Transcriptome and metabolome analysis

The widely targeted metabolome analysis and RNA sequencing were carried outby Wuhan Metware Biotechnology Co., Ltd (Metware Biotechnology, China). The analysis of metabolic pathways was perpformed by using the KEGG metabolic pathway database. A threshold for factors of variable importance in projection (VIP) ≥ 1 and fold change ≥2 or ≤ 0.5 was established for metabolites with significant differences in content. pGWB18-Myc-*SmMYB36*-transformed hairy roots were the experimental sample and ATCC15834-induced hairy roots were the control sample. Three replicates of each sample.

All clean reads were used with HISAT2 to map to the reference genome of *S. miltiorrhiza* [34]. Transcript abundance and differentially expressed genes (DEGs) were analysed identically to as previously described [33]. All DEGs were annotated according to GO; KEGG; KOG; Swiss-Prot; TrEMBL databases.

### Electrophoretic mobility shift assays (EMSA)

HIS protein and recombinant protein His-SmMYB36 were purified as reported before [32]. Analysis of the 2000 bp promoter region upstream of the start codon ATG of *SmGAPC*, *SmRAS*, *SmDXS2*, *SmGGPPS1*, *SmCPS1*, *SmERF6*, and *SmERF115* using the plantcare online site revealed core sequences containing MBSI and MBSII. Then, a 48 bp sequence containing the promoter fragment of the MBSI 3 × (CNGTT(G/A)) and 3 × MBSII (TNGTT(G/A)) core sequences was used as a probe, and the mutant MBSI 3 × (AAAAAA) and MBSII 3 × (AAAAAA) core sequences were used as control probes. The single-stranded probe was synthesized by Sangon Biotech, and only the biotin marker was added at the end of the 3′ end of the sense strand, and no marker was added to the complementary strand. The solution was aspirated at F chain: R chain = 1:1, mixed well in the centrifuge tube, and placed in a PCR instrument for annealing at 90°C for 10 min, and then slowly cooled down to room temperature to obtain a double-stranded probe master mix at a concentration of 10 μM. Follow-up experiments were performed according to the instructions of the LightShift EMSA Optimization and Control Kit (Thremo, China).

### Dual-luciferase (dual-LUC) assays

Experiments with dual-LUC were conducted as previously described [35]. Different combinations of reporter and effector plasmids were co-transformed transiently into four-week-old tobacco (*Nicotiana benthamiana*) leaves. GloMax20/20 luminometers (Promega, USA) instrument and dual-Luciferase® Reporter Assay System were used to determine the results. The results were performed in three biological replicates.

### Data analysis

Data were processed with SPSS26.0 software, and one-way ANOVA and Duncan’s multiple range test were used for significance analysis between three or more groups of samples, and Student’s *t*-test was used for significance analysis between two groups of samples.

## Acknowledgements

We thank Ningjuan Fan, Xiyan Chen and Hui Duan of the Center for Teaching and Research Equipment, College of Life Sciences, Northwest A&F University for their technical support. This work was supported by the National Natural Science Foundation of China (Project No. 32270278).

## Author contributions

P.M. and J.D. conceived the idea of the review and made the final modification. Q.L., X.F., and Y.Z. performed the experiments and data processing. R.C. provided assistance in the qPCR experiments. Q.L. and P.M. wrote the manuscript. All authors approved the final manuscript.

## Data availability

Detailed data supporting the findings of this study can be found in the supplementary material.

## Conflict of interest

The authors have no conflicts of interest to declare.

## Supplementary data

Supplementary data is available at *Horticulture Research* online.

## Supplementary Material

Web_Material_uhac238

## References

[1] Liu J , Osbourn A, Ma P. MYB transcription factors as regulators of phenylpropanoid metabolism in plants. *Mol Plant.* 2015;8:689–708. 25840349 10.1016/j.molp.2015.03.012

[2] Ma D , Constabel CP. MYB repressors as regulators of phenylpropanoid metabolism in plants. *Trends Plant Sci.* 2019;24:275–89. 30704824 10.1016/j.tplants.2018.12.003

[3] Tholl D . Biosynthesis and biological functions of terpenoids in plants. *Adv Biochem Eng Biotechnol.* 2015;148:63–106. 25583224 10.1007/10_2014_295

[4] Mahjoub A , Hernould M, Joubès J et al. Overexpression of a grapevine R2R3-MYB factor in tomato affects vegetative development, flower morphology and flavonoid and terpenoid metabolism. *Plant Physiol Biochem.* 2009;47:551–61. 19375343 10.1016/j.plaphy.2009.02.015

[5] Zhu F , Luo T, Liu C et al. An R2R3-MYB transcription factor represses the transformation of α- and β-branch carotenoids by negatively regulating expression of *CrBCH2* and *CrNCED5* in flavedo of citrus reticulate. *New Phytol.* 2017;216:178–92. 28681945 10.1111/nph.14684

[6] Zvi MMB , Shklarman E, Masci T et al. PAP1 transcription factor enhances production of phenylpropanoid and terpenoid scent compounds in rose flowers. *New Phytol.* 2012;195:335–45. 22548501 10.1111/j.1469-8137.2012.04161.x

[7] Bedon F , Bomal C, Caron S et al. Subgroup 4 R2R3-MYBs in conifer trees: gene family expansion and contribution to the isoprenoid- and flavonoid-oriented responses. *J Exp Bot.* 2010;61:3847–64. 20732878 10.1093/jxb/erq196 PMC2935864

[8] Ma P , Liu J, Osbourn A et al. Regulation and metabolic engineering of tanshinone biosynthesis. *RSC Adv.* 2015;5:18137–44.

[9] Ma P , Liu J, Zhang C et al. Regulation of water-soluble phenolic acid biosynthesis in *salvia miltiorrhiza* Bunge. *Appl Biochem Biotechnol.* 2013;170:1253–62. 23673485 10.1007/s12010-013-0265-4

[10] Wu SJ , Shi M, Wu JY. Cloning and characterization of the 1-deoxy-D-xylulose 5-phosphate reductoisomerase gene for diterpenoid tanshinone biosynthesis in *salvia miltiorrhiza* (Chinese sage) hairy roots. *Biotechnol Appl Biochem.* 2009;52:89–95. 18302535 10.1042/BA20080004

[11] Kai G , Xu H, Zhou C et al. Metabolic engineering tanshinone biosynthetic pathway in *salvia miltiorrhiza* hairy root cultures. *Metab Eng.* 2011;13:319–27. 21335099 10.1016/j.ymben.2011.02.003

[12] Dai Z , Cui G, Zhou SF et al. Cloning and characterization of a novel 3-hydroxy-3-methylglutaryl coenzyme a reductase gene from salvia miltiorrhiza involved in diterpenoid tanshinone accumulation. *J Plant Physiol.* 2011;168:148–57. 20637524 10.1016/j.jplph.2010.06.008

[13] Zhang X , Guan H, Dai Z et al. Functional analysis of the isopentenyl diphosphate isomerase of *salvia miltiorrhiza* via color complementation and RNA interference. *Molecules.* 2015;20:20206–18. 26569204 10.3390/molecules201119689 PMC6332163

[14] Cui G , Duan L, Jin B et al. Functional divergence of diterpene syntheses in the medicinal plant *salvia miltiorrhiza*. *Plant Physiol.* 2015;169:1607–18. 26077765 10.1104/pp.15.00695 PMC4634056

[15] Guo J , Zhou YJ, Hillwig ML et al. CYP76AH1 catalyzes turnover of miltiradiene in tanshinones biosynthesis and enables heterologous production of ferruginol in yeasts. *Proc Natl Acad Sci U S A.* 2013;110:12108–13. 23812755 10.1073/pnas.1218061110 PMC3718111

[16] Guo J , Ma X, Cai Y et al. Cytochrome P450 promiscuity leads to a bifurcating biosynthetic pathway for tanshinones. *New Phytol* 2016;210:525–34. 26682704 10.1111/nph.13790 PMC4930649

[17] Li B , Li J, Chai Y et al. Targeted mutagenesis of *CYP76AK2* and *CYP76AK3* in *salvia miltiorrhiza* reveals their roles in tanshinones biosynthetic pathway. *Int J Biol Macromol.* 2021;189:455–63. 34419551 10.1016/j.ijbiomac.2021.08.112

[18] Hu Z , Ren L, Bu J et al. Functional characterization of a 2OGD involved in abietane-type diterpenoids biosynthetic pathway in *salvia miltiorrhiza*. *Front Plant Sci* 2022;13:947674. 35873989 10.3389/fpls.2022.947674 PMC9301305

[19] Song S , Qi T, Huang H et al. The Jasmonate-ZIM domain proteins interact with the R2R3-MYB transcription factors MYB21 and MYB24 to affect Jasmonate-regulated stamen development in *Arabidopsis*. *Plant Cell.* 2011;23:1000–13. 21447791 10.1105/tpc.111.083089 PMC3082250

[20] Di P , Zhang L, Chen JF et al. ^13^C tracer reveals phenolic acids biosynthesis in hairy root cultures of *salvia miltiorrhiza*. *ACS Chem Biol.* 2013;8:1537–48. 23614461 10.1021/cb3006962

[21] Sun M , Shi M, Wang Y et al. The biosynthesis of phenolic acids is positively regulated by the JA-responsive transcription factor ERF115 in *salvia miltiorrhiza*. *J Exp Bot.* 2019;70:243–54. 30299490 10.1093/jxb/ery349

[22] Bai Z , Li W, Jia Y et al. The ethylene response factor SmERF6 co-regulates the transcription of *SmCPS1* and *SmKSL1* and is involved in tanshinone biosynthesis in *salvia miltiorrhiza* hairy roots. *Planta.* 2018;248:243–55. 29704055 10.1007/s00425-018-2884-z

[23] Hao X , Pu Z, Cao G et al. Tanshinone and salvianolic acid biosynthesis are regulated by in hairy roots. *J Adv Res* 2020;23:1–12. 32071787 10.1016/j.jare.2020.01.012 PMC7016019

[24] Zhang J , Zhou L, Zheng X et al. Overexpression of *SmMYB9b* enhances tanshinone concentration in *salvia miltiorrhiza* hairy roots. *Plant Cell Rep.* 2017;36:1297–309. 28508121 10.1007/s00299-017-2154-8

[25] Liu L , Song S, Li D et al. SmMYB98b positive regulation to tanshinones in *salvia miltiorrhiza* Bunge hairy roots. *Plant Cell Tissue Organ Cult.* 2019;140:459–67.

[26] Deng C , Wang Y, Huang F et al. SmMYB2 promotes salvianolic acid biosynthesis in the medicinal herb *salvia miltiorrhiza*. *J Integr Plant Biol.* 2020;62:1688–702. 32343491 10.1111/jipb.12943

[27] Li S , Wu Y, Kuang J et al. SmMYB111 is a key factor to phenolic acid biosynthesis and interacts with both SmTTG1 and SmbHLH51 in *salvia miltiorrhiza*. *J Agric Food Chem.* 2018;66:8069–78. 30001627 10.1021/acs.jafc.8b02548

[28] Zhou W , Shi M, Deng C et al. The methyl jasmonate-responsive transcription factor SmMYB1 promotes phenolic acid biosynthesis in *salvia miltiorrhiza*. *Hortic Res.* 2021;8:1–13. 33384411 10.1038/s41438-020-00443-5 PMC7775463

[29] Hao G , Jiang X, Feng L et al. Cloning, molecular characterization and functional analysis of a putative R2R3-MYB transcription factor of the phenolic acid biosynthetic pathway in s. miltiorrhiza bge. f. Alba. *Plant Cell Tissue Organ Cult.* 2015;124:151–68.

[30] Li L , Wang D, Zhou L et al. JA-responsive transcription factor SmMYB97 promotes phenolic acid and tanshinone accumulation in *salvia miltiorrhiza*. *J Agric Food Chem.* 2020;68:14850–62. 33284615 10.1021/acs.jafc.0c05902

[31] Zhang S , Ma P, Yang D et al. Cloning and characterization of a putative R2R3 MYB transcriptional repressor of the rosmarinic acid biosynthetic pathway from *salvia miltiorrhiza*. *PLoS One.* 2013;8:e73259. 24039895 10.1371/journal.pone.0073259 PMC3769309

[32] Ding K , Pei T, Bai Z et al. SmMYB36, a novel R2R3-MYB transcription factor, enhances tanshinone accumulation and decreases phenolic acid content in *salvia miltiorrhiza* hairy roots. *Sci Rep.* 2017;7:5104. 28698552 10.1038/s41598-017-04909-w PMC5506036

[33] Pei T , Ma P, Ding K et al. SmJAZ8 acts as a core repressor regulating JA-induced biosynthesis of salvianolic acids and tanshinones in *salvia miltiorrhiza* hairy roots. *J Exp Bot.* 2018;69:1663–78. 29281115 10.1093/jxb/erx484

[34] Xu H , Song J, Luo H et al. Analysis of the genome sequence of the medicinal plant *salvia miltiorrhiza*. *Mol Plant.* 2016;9:949–52. 27018390 10.1016/j.molp.2016.03.010 PMC5517341

[35] Huang Q , Sun M, Yuan T et al. The AP2/ERF transcription factor SmERF1L1 regulates the biosynthesis of tanshinones and phenolic acids in *salvia miltiorrhiza*. *Food Chem.* 2019;274:368–75. 30372953 10.1016/j.foodchem.2018.08.119

[36] Yoshida Y , Sano R, Wada T et al. Jasmonic acid control of GLABRA3 links inducible defense and trichome patterning in Arabidopsis. *Development.* 2009;136:1039–48. 19234066 10.1242/dev.030585

[37] Urao T , Noji MA, Yamaguchi-Shinozaki K et al. A transcriptional activation domain of ATMYB2, a drought-inducible Arabidopsis Myb-related protein. *Plant J.* 1996;10:1145–8. 9011094 10.1046/j.1365-313x.1996.10061145.x

[38] Kagale S , Rozwadowski K. EAR motif-mediated transcriptional repression in plants: an underlying mechanism for epigenetic regulation of gene expression. *Epigenetics.* 2011;6:141–6. 20935498 10.4161/epi.6.2.13627 PMC3278782

[39] Qi T , Song S, Ren Q et al. The Jasmonate-ZIM-domain proteins interact with the WD-repeat/bHLH/MYB complexes to regulate Jasmonate-mediated anthocyanin accumulation and trichome initiation in *Arabidopsis thaliana*. *Plant Cell.* 2011;23:1795–814. 21551388 10.1105/tpc.111.083261 PMC3123955

[40] Zhou M , Zhang K, Sun Z et al. LNK1 and LNK2 corepressors interact with the MYB3 transcription factor in phenylpropanoid biosynthesis. *Plant Physiol.* 2017;174:1348–58. 28483877 10.1104/pp.17.00160 PMC5490896

[41] Tang M , Li B, Zhou X et al. A genome-scale TF-DNA interaction network of transcriptional regulation of Arabidopsis primary and specialized metabolism. *Mol Syst Biol.* 2021;17:e10625. 34816587 10.15252/msb.202110625 PMC8611409

[42] Tzin V , Malitsky S, Zvi MMB et al. Expression of a bacterial feedback-insensitive 3-deoxy-D-arabino- heptulosonate 7-phosphate synthase of the shikimate pathway in Arabidopsis elucidates potential metabolic bottlenecks between primary and secondary metabolism. *New Phytol.* 2012;194:430–9. 22296303 10.1111/j.1469-8137.2012.04052.x

[43] Tzin V , Rogachev I, Meir S et al. Tomato fruits expressing a bacterial feedback-insensitive 3-deoxy-D-arabino- heptulosonate 7-phosphate synthase of the shikimate pathway possess enhanced levels of multiple specialized metabolites and upgraded aroma. *J Exp Bot.* 2013;64:4441–52. 24006429 10.1093/jxb/ert250 PMC3808321

[44] Zhang Y , Butelli E, Alseekh S et al. Multi-level engineering facilitates the production of phenylpropanoid compounds in tomato. *Nat Commun.* 2015;6:8635. 26497596 10.1038/ncomms9635 PMC4639801

[45] Ying S , Su M, Wu Y et al. Trichome regulator SlMIXTA-like directly manipulates primary metabolism in tomato fruit. *Plant Biotechnol J.* 2020;18:354–63. 31254436 10.1111/pbi.13202 PMC6953195

[46] Zhao H , Zhong S, Sang L et al. PaACL silencing accelerates flower senescence and changes the proteome to maintain metabolic homeostasis in Petunia hybrida. *J Exp Bot.* 2020;71:4858–76. 32364241 10.1093/jxb/eraa208 PMC7475263

[47] Zhao H , Chen G, Sang L et al. Mitochondrial citrate synthase plays important roles in anthocyanin synthesis in petunia. *Plant Sci.* 2021;305:110835. 33691969 10.1016/j.plantsci.2021.110835

[48] Rius SNP , Casati P, Iglesias AA et al. Characterization of Arabidopsis lines deficient in GAPC-1, a cytosolic NAD-dependent glyceraldehyde-3-phosphate dehydrogenase. *Plant Physiol.* 2008;148:1655–67. 18820081 10.1104/pp.108.128769 PMC2577239

[49] Yu H , Gerstein M. Genomic analysis of the hierarchical structure of regulatory networks. *Proc Natl Acad Sci U S A.* 2006;103:14724–31. 17003135 10.1073/pnas.0508637103 PMC1595419

[50] McCarthy RL , Zhong R, Ye Z-H. MYB83 is a direct target of SND1 and acts redundantly with MYB46 in the regulation of secondary cell wall biosynthesis in Arabidopsis. *Plant Cell Physiol.* 2009;50:1950–64. 19808805 10.1093/pcp/pcp139

[51] Zhong R , Ye Z-H. MYB46 and MYB83 bind to the SMRE sites and directly activate a suite of transcription factors and secondary wall biosynthetic genes. *Plant Cell Physiol.* 2012;53:368–80. 22197883 10.1093/pcp/pcr185

[52] Ko JH , Jeon HW, Kim WC et al. The MYB46/MYB83-mediated transcriptional regulatory programme is a gatekeeper of secondary wall biosynthesis. *Ann Bot.* 2014;114:1099–107. 24984711 10.1093/aob/mcu126 PMC4195559

[53] Chen M , Yan T, Shen Q et al. GLANDULAR TRICHOME-SPECIFIC WRKY 1 promotes artemisinin biosynthesis in *Artemisia annua*. *New Phytol.* 2017;214:304–16. 28001315 10.1111/nph.14373

[54] Mangan S , Alon U. Structure and function of the feed-forward loop network motif. *Proc Natl Acad Sci U S A.* 2003;100:11980–5. 14530388 10.1073/pnas.2133841100 PMC218699

[55] Zhou Y , Feng J, Li Q et al. MYC2b enhances tanshinone accumulation in by activating pathway genes and promoting lateral root development. *Front Plant Sci.* 2020;11:559438. 33042182 10.3389/fpls.2020.559438 PMC7517298

[56] Zhou Y , Sun W, Chen J et al. SmMYC2a and SmMYC2b played similar but irreplaceable roles in regulating the biosynthesis of tanshinones and phenolic acids in *salvia miltiorrhiza*. *Sci Rep.* 2016;6:22852. 26947390 10.1038/srep22852 PMC4780012

[57] Yang N , Zhou W, Su J et al. Overexpression of increases the production of phenolic acids in *salvia miltiorrhiza*. *Front Plant Sci.* 2017;8:1804. 29230228 10.3389/fpls.2017.01804 PMC5708653

[58] Li W , Bai Z, Pei T et al. SmGRAS1 and SmGRAS2 regulate the biosynthesis of tanshinones and phenolic acids in *salvia miltiorrhiza*. *Front Plant Sci.* 2019;10:1367. 31737003 10.3389/fpls.2019.01367 PMC6831727

[59] Deng C , Shi M, Fu R et al. ABA-responsive transcription factor bZIP1 is involved in modulating biosynthesis of phenolic acids and tanshinones in *salvia miltiorrhiza*. *J Exp Bot.* 2020;71:5948–62. 32589719 10.1093/jxb/eraa295

[60] Zhang C , Xing B, Yang D et al. SmbHLH3 acts as a transcription repressor for both phenolic acids and tanshinone biosynthesis in *salvia miltiorrhiza* hairy roots. *Phytochemistry.* 2020;169:112183. 31704239 10.1016/j.phytochem.2019.112183

