# Supplementary figures and images for: The SmMYB36-SmERF6/SmERF115 module regulates the biosynthesis of tanshinones and phenolic acids in salvia miltiorrhiza hairy roots
Source: Hortic Res. 2022 Oct 26;10(1):uhac238. doi: 10.1093/hr/uhac238 (PMC9832864; doi:10.1093/hr/uhac238)

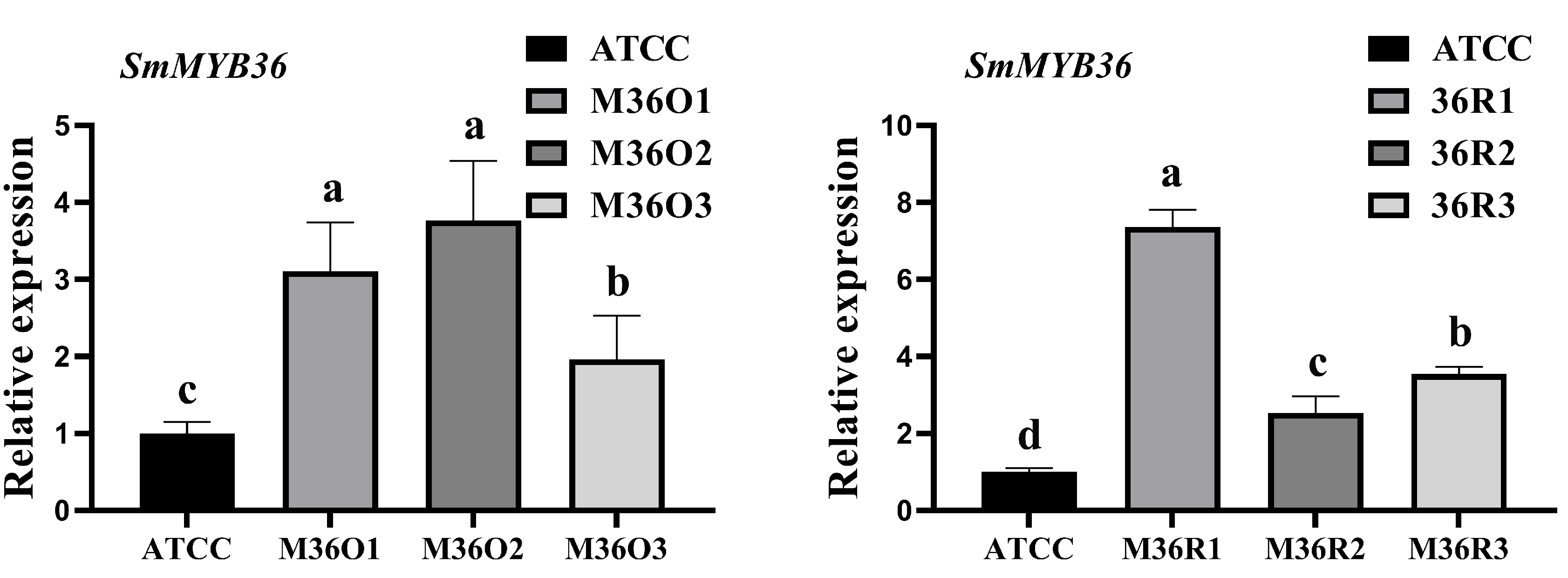

Supplement: Web_Material_uhac238 [file web_material_uhac238.zip › Fig.S2.tif]

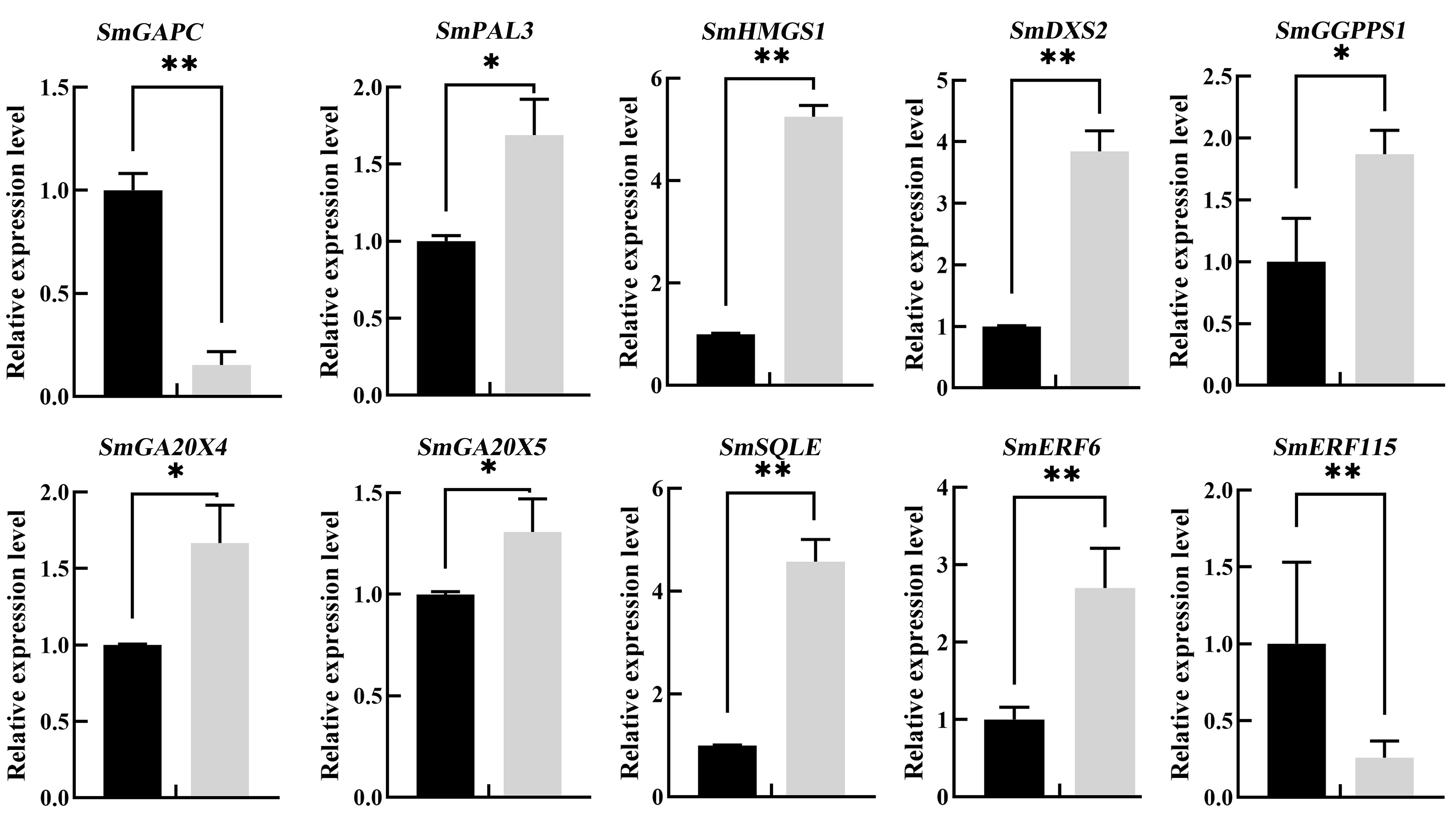

Supplement: Web_Material_uhac238 [file web_material_uhac238.zip › Fig.S3.tif]

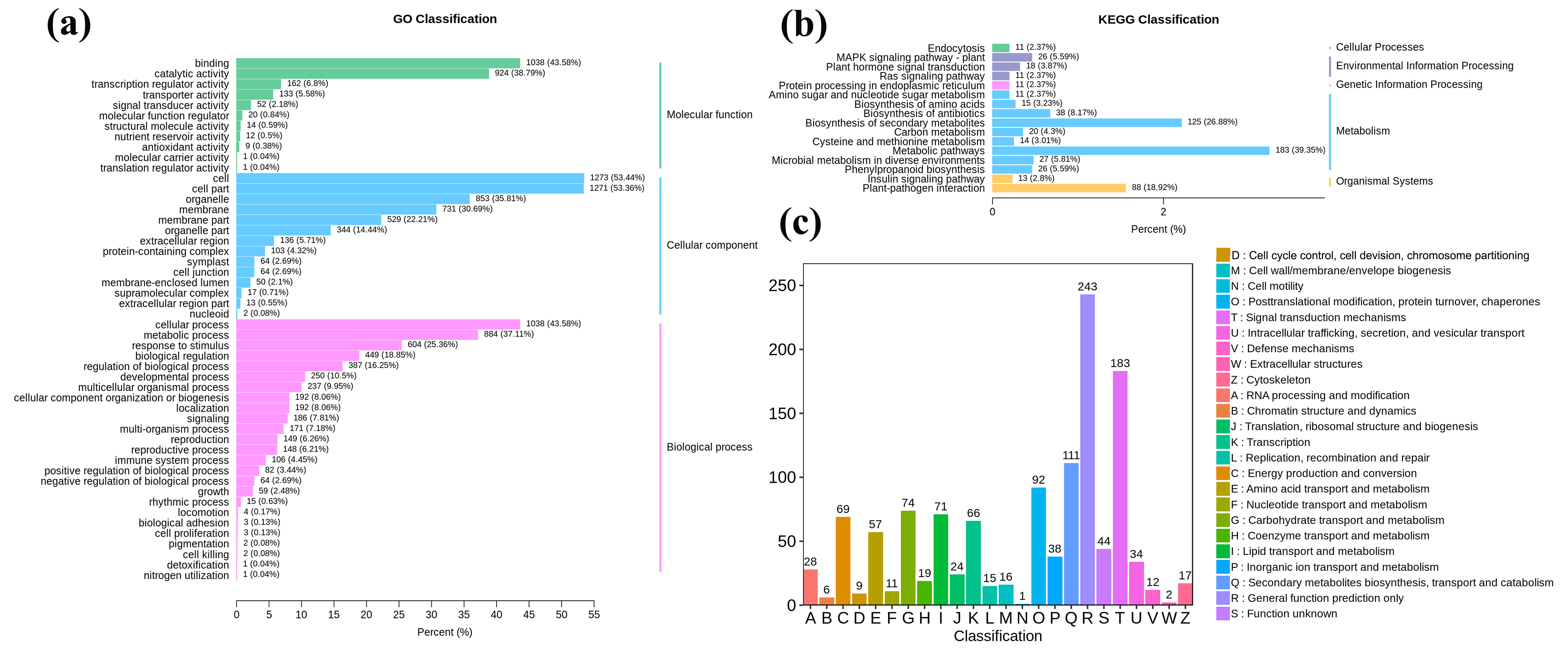

Supplement: Web_Material_uhac238 [file web_material_uhac238.zip › Fig.S4.tif]

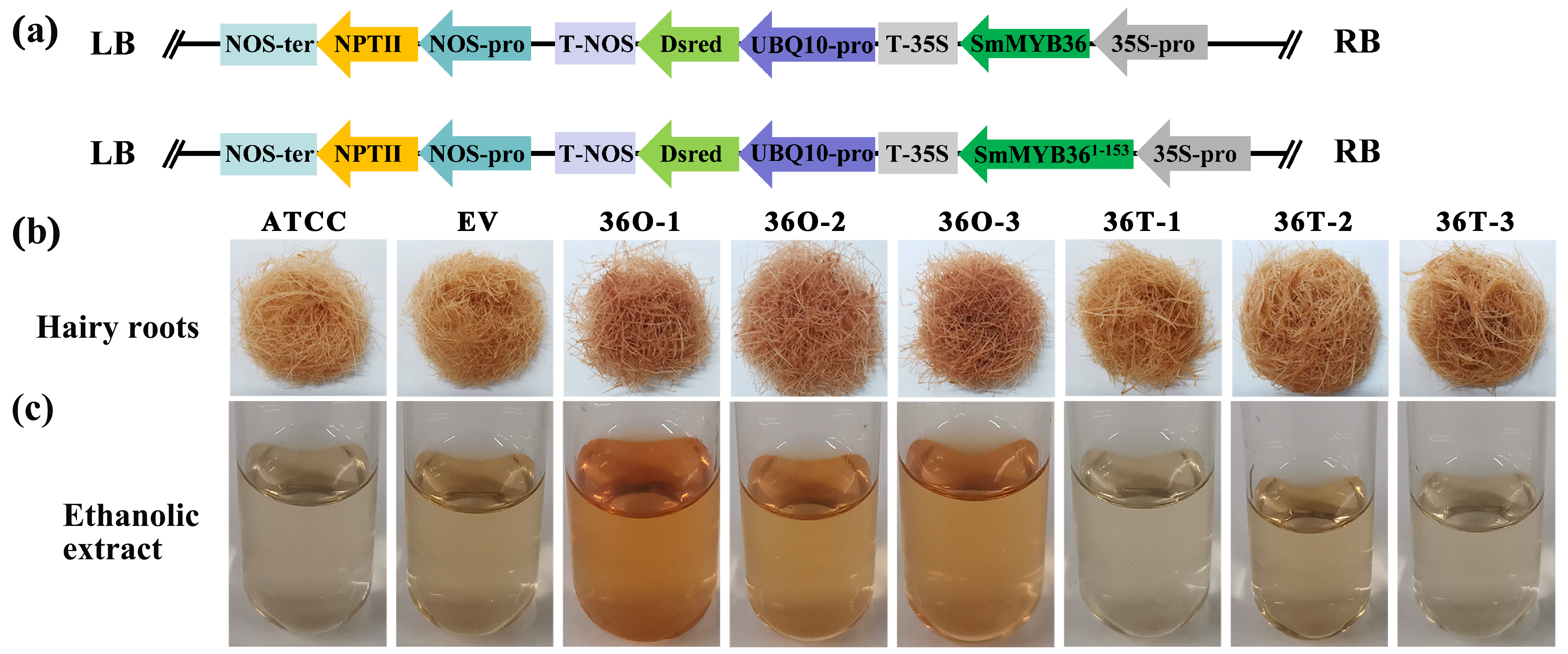

Supplement: Web_Material_uhac238 [file web_material_uhac238.zip › Fig.S5.tif]

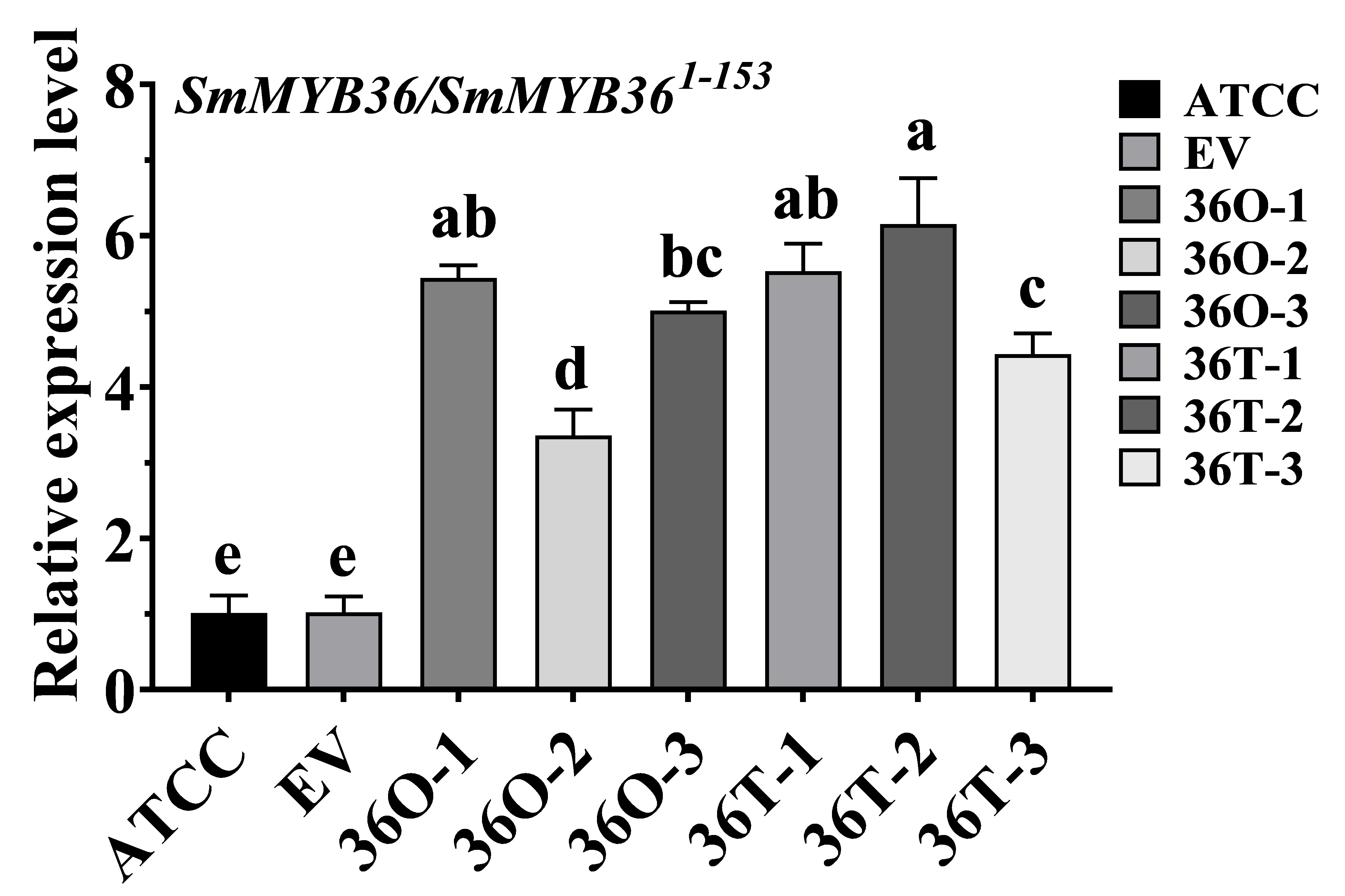

Supplement: Web_Material_uhac238 [file web_material_uhac238.zip › Fig.S6.tif]

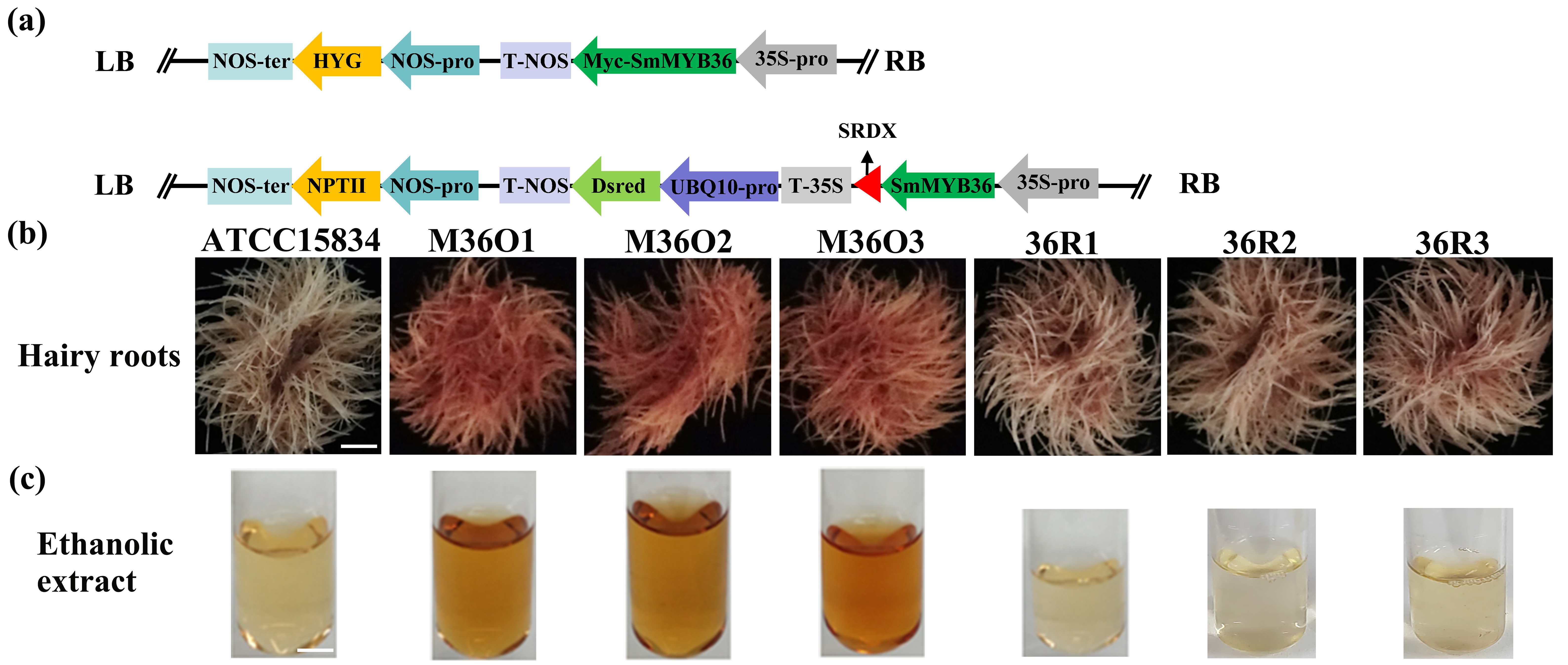

Supplement: Web_Material_uhac238 [file web_material_uhac238.zip › FigS1.tif]
